# Economic evaluation of the artificial liver support system MARS in patients with acute-on-chronic liver failure

**DOI:** 10.1186/1478-7547-4-16

**Published:** 2006-10-05

**Authors:** Franz P Hessel

**Affiliations:** 1 Institute for Health Care Management, University of Duisburg-Essen, Campus Essen, Schützenbahn, D-45127 Essen, Germany

## Abstract

**Background:**

Acute-on-chronic liver failure (ACLF) is a life threatening acute decompensation of a pre-existing chronic liver disease. The artificial liver support system MARS is a new emerging therapeutic option possible to be implemented in routine care of these patients. The medical efficacy of MARS has been demonstrated in first clinical studies, but economic aspects have so far not been investigated. Objective of this study was to estimate the cost-effectiveness of MARS.

**Methods:**

In a clinical cohort trial with a prospective follow-up of 3 years 33 ACLF-patients treated with MARS were compared to 46 controls. Survival, health-related quality of life as well as direct medical costs for in- and outpatient treatment from a health care system perspective were determined. Based on the differences in outcome and indirect costs the cost-effectiveness of MARS expressed as incremental costs per life year gained and incremental costs per QALY gained was estimated.

**Results:**

The average initial intervention costs for MARS were 14600 EUR per patient treated. Direct medical costs over 3 years follow up were overall 40000 EUR per patient treated with MARS respectively 12700 EUR in controls. The 3 year survival rate after MARS was 52% compared to 17% in controls. Kaplan-Meier analysis of cumulated survival probability showed a highly significant difference in favour of MARS. Incremental costs per life-year gained were 31400 EUR; incremental costs per QALY gained were 47200 EUR.

**Conclusion:**

The results after 3 years follow-up of the first economic evaluation study of MARS based on empirical patient data are presented. Although high initial treatment costs for MARS occur the significantly better survival seen in this study led to reasonable costs per live year gained. Further randomized controlled trials investigating the medical efficacy and the cost-effectiveness are recommended.

## Background

Acute-on-chronic liver failure (ACLF) is a sudden, severe, life-threatening deterioration of the liver function in patients with a chronic pre-existing liver disease. The prevalence of chronic liver disease in Germany is estimated to lie between 0.5 and 1%[1]. The most frequent underlying reason for ACLF is an irreversible liver damage due to chronic alcohol abuse, followed by viral hepatitis (e.g. HBV, HCV) and autoimmune disorders[2].

Characteristic complications of ACLF are the development of a renal dysfunction and a multiorgan failure, arterial hypotension, disseminated intravascular coagulation or a disorder of microcirculation leading to damage of extrahepatic organs. All therapeutic measures aim at the stabilization of the liver function until an improvement of the symptoms by the self-regeneration of the liver has been achieved or until a suitable organ for liver transplantation is available (bridging to transplantation)[3]. The 30d-mortality of patients with ACLF is high[2], but the liver has a potential to regenerate. If the patients survive the first crucial weeks of the acute decompensation the 5-year survival rate is relatively high.

Conventional diagnostic procedures and therapy of ACLF focus on to identify triggering events and reasons of liver failure like viral infection, alcohol abuse or acute intoxication, and to avoid them. Further objectives are to prevent the development respectively the progression of secondary organ dysfunctions or organ failure (like heart or renal failure or cerebral complications).

Under certain circumstances a liver transplantation is an option for the therapy of liver failure, but the main limiting factor is the availability of suitable organs. Furthermore in many countries a present chronic alcohol abuse is a contra-indication for placement on a liver transplantation list. The initial health state and the rapidness of the progression of the disease are the most important prognostic factors for a successful transplantation[3].

The endocrinological function of the liver can at least partly be compensated, but the removal of toxins can only marginally be substituted by conventional conservative therapy. To improve this important component of the liver function is the main objective of artificial extracorporal liver support systems[4]. A number of systems have been developed but none of them was successful in being implemented in routine care of patients with liver failure so far. The currently most widespread system, the **M**olecular **A**dsorbent **R**ecirculating **S**ystem (MARS) is used in a number of specialized centres in addition to standard care. It was developed during the 1990s and is based on a modified albumin dialysis system [5,6].

The technology has been on the market since 1999 and since this time a few thousand patients, most of them with an ACLF were treated worldwide [7-9]. Until 2003 there was no regular reimbursement of artificial liver support technologies and the use of the systems was paid in the context of industry financed studies or by the hospitals as part of their individual budgets. In 2004 a category "extracorporal liver assist device" was included in the list of the so-called "additional payments" (Zusatzentgelte) in the German DRG-system. This allows a limited reimbursement for hospitals using the technology in inpatient care. The reimbursement rate as well as the number of patients has to be negotiated between the hospital and the statutory sickness funds[10].

The clinical efficacy of MARS has been demonstrated in several randomized controlled trials. Significant advantages have been shown concerning survival after 30 days, the extension of hepatic encephalopathy, hemodynamic parameter and some laboratory tests of liver function[11,12]. Except the completely uncontrolled annual reports of the MARS registry[7] there are no published empirical studies on long-time survival, costs or cost-effectiveness.

Preliminary results of a pilot study of the Rostock controlled clinical cohort trial of the treatment of patients with liver failure were published previously[13,14]. Presented here are the results after a follow-up of 3 years.

The objectives were to determine the outcomes survival rates, survival hazard ratios, and mean survival time, the direct medical costs for acute treatment of ACLF and follow-up, and the incremental cost-outcome ratios expressed in costs per life-years gained and costs per QALY over three years in patients with ACLF and an underlying alcoholic liver disease.

## Methods

### Study design and population

In a prospective clinical cohort study the influence on survival and direct medical costs of the treatment of patients with an ACLF and a documented previous alcohol abuse with the artificial liver support system MARS over a period of three years was investigated. To estimate the cost-effectiveness of MARS the incremental costs per life-year gained and the incremental costs per QALY gained were determined.

The study population consists of all adult patients admitted to the Rostock university hospital for internal medicine for at least seven days with an acute deterioration of a previously documented alcoholic liver disease or a previously known chronic liver disease and a documented alcohol abuse for more than five years. Further inclusion criteria was a total bilirubin higher than 300 mmol/μl at any time during the hospital stay. Exclusion criteria were severe gastrointestinal bleeding, carcinoma or other severe co-morbidity and placement on liver transplant list.

All patients between 1999 and the beginning of 2003 were recruited consecutively.

Patients of the intervention group were treated with the standardized MARS kit (Teraklin^®^) about 9 days (8.7d, SD 2.8d) after admission with approximately 5 courses (5.4 courses, SD 1.7 courses) of MARS on consecutive days additionally to standard conservative treatment of liver failure. Patients of the control group received only standard treatment including every therapeutic option except MARS. In both groups the patients were treated with the heterogenous standard repertoire of pharmaceutical and medical options including also costly procedures like renal dialysis, blood products and ICU care. Except MARS there was no systematic difference in treatment of the two groups of patients concerning the principles of standard treatment. Informed consent of all participating patients was taken and the data collection and analysis was in line with the principles outlined by the local ethic committee.

### Acquisition of data

Patient's data files and hospital's internal statistics of resource uses, single item costs or if appropriate package costs (e.g. overheads per day) were used to extract information about the inpatient hospital stay in the Rostock university hospital. Resource use, health-related quality of life, and clinical outcomes during the follow-up were documented using annually performed standardized telephone interviews and written questionnaires for patients, GPs and hospitals.

As published previously in detail health-related quality of life of the surviving patients was measured with the German versions of the EQ-5D and SF-12[13].

All costs and all survival times were standardized to a three year follow-up period. Thus patients' careers from admittance to Rostock university hospital to either December 2005 or the death of the patient were drawn. For validation of the determined survival time and minimization of missing values the regional residents' registration offices were contacted.

### Determination of costs

Direct medical costs were calculated from a health care system's perspective approximated by the actual hospital cost, market prices of drugs and medical devices and average single item reimbursement rates for a weighted mix of members of private and statutory sickness funds[15]. Direct non-medical costs and indirect costs were not included. The healthcare system's perspective was chosen instead of a payer's perspective to take into consideration the fact that MARS was not reimbursed by German sickness funds before 2004 when the patients were treated and therefore relevant intervention costs would have been missed from a pure payer's perspective.

All resource uses were valuated with year 2002 prices and standardized to EUR of year 2002 without discounting effects or costs in the basecase analysis. Cost calculations were performed according to national and international guidelines for economic evaluation of health care techniques [15-19].

### Statistical methods

Mortality rates after one, two and three years were calculated and a Kaplan Meier analysis for the period of three years was performed to consider also the variation of mortality over time. The significance of the difference in cumulative survival was tested with Log rank[20]. The difference in baseline characteristics was tested with one-way ANOVA. The influence of clinical and socio-demographic parameter on direct medical costs was tested with OLS-regression respectively Mann-Whitney-U-Test for dichotomous variables. For all statistical analyses the standard software packages SPSS 12.0 and SAS were used.

## Results

Overall 79 patients with an alcohol-induced ACLF could be identified according to the described inclusion and exclusion criteria. 33 of these patients were treated with MARS; the other 46 patients were not treated with an artificial liver support system and consequently defined as control group. Of all screened patients overall four patients (two of each group) considered for liver transplantation had been excluded.

All 79 patients meeting the inclusion and exclusion criteria could be enrolled in the study and for 100% of the patients' baseline characteristics, resource use and survival time could be determined. For 87% (MARS: 29; controls: 40) of the patients health related quality of life and resource uses could be documented completely. Two patients (one of each group) refused to answer questions about their quality of life and in three patients (MARS: 1; Controls: 2) for at least one follow-up time point the individual health state did not allow answering the questionnaires personally. In two cases relatives could be contacted instead. For five patients a part of the data on inpatient resource use was missing due to incomplete patient files.

### Baseline characteristics

The groups did not differ statistically significant in age, sex, severity of ACLF (expressed in CHILD-score value), platelet count and peak value of total bilirubin. The MARS group had a slightly higher percentage of women and slightly higher platelet counts and maximum of total bilirubin but these differences were not considered to be clinically relevant for outcome or costs.

### Survival

For all 79 included patients the survival time could be determined over a period of 3 years after treatment of ACLF. Nearly 2 out of 3 patients could be discharged alive after hospital treatment with a slightly higher percentage in the MARS group. After one year 58% of the patients of the MARS group were alive compared to 35% of the controls. The survival rates after a follow-up of 3 years were 52% in the MARS group and 17% in the control group. The exact numbers are shown in Table 2.

**Table 1:** Baseline characteristics

	**MARS**	**Controls**	**p-value**
Sample size	33	46	–
Age (years, Mean ± SD)	47.2 (± 10.54)	48.5 (± 13.05)	.655
Sex male/female, n (%)	19/14 (58/42)	31/15 (67/33)	.379
Child score (Mean ± SD)	11.4 (± 1.77)	11.7 (± 1.66)	.453
Platelets (Mean ± SD)	138 (118)	116 (98)	.378
Bilirubin total (Mean ± SD)	487 (144)	440 (97)	.185

**Table 2:** Survival rates

	**After Hospital**	**After 1 year**	**After 2 years**	**After 3 years**
**MARS (n = 33)**	n = 22/67%	N = 19/58%	n = 17/52%	n = 17/52%
**Controls (n = 46)**	n = 29/63%	N = 16/35%	n = 12/26%	n = 8/17%
**Total (n = 79)**	n = 51/63%	N = 35/44%	n = 29/37%	n = 25/32%

**Figure 1:**
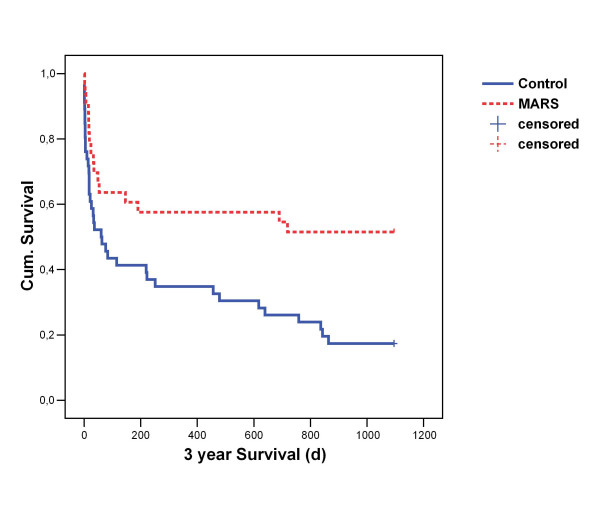
Kaplan Meier Analysis after 3 years follow-up.

The mean survival time after 3 years follow-up was 624d of possible 1095d in the MARS group and 339d in the control group. The difference in cumulative survival probability using Kaplan Meier analysis was highly significant on a p = 0,05 level tested with Logrank statistics (p = 0,0035). Figure 1 shows the Kaplan Meier survival curves.

### Direct medical costs for acute care inpatient treatment

ACLF patients treated with MARS had more (Mean 33d, SD 18d) inpatient hospital days compared to controls (Mean 21d, SD 10). This trend could be observed also for the number of days in the ICU (MARS 4d, SD 8d; Controls 1d; SD 4d).

The total costs for acute inpatient treatment (costs for MARS included) were higher in the MARS group with mean costs per patient of 31539 EUR (SD 19618 EUR) compared to controls with 7543 EUR (SD 6527 EUR). MARS itself was responsible for 14631 EUR per patient. The remaining difference between the two groups after excluding the costs for MARS is mainly caused by the higher number of hospital days and a larger amount of blood products in the MARS group. The detailed distribution of the hospital's costs is shown in Table 3.

**Table 3:** Direct medical costs for acute care treatment of ACLF in EUR 2002

	**MARS**		**Controls**	
	Mean	SD	Mean	SD
Physician Non-ICU	760	555	509	295
Nursing Non-ICU	1332	974	892	517
Medical Care Non-ICU	959	644	824	1663
Physician ICU	366	843	124	352
Nursing ICU	1261	2909	427	1215
Medical Care ICU	2375	5478	804	2288
Drugs	1363	1960	503	665
Blood products	4086	5279	870	1896
Lab-tests	1488	812	868	569
MARS	14631	7419	0	-
Overheads	2718	1483	1722	857

**Total**	**31539**	**19681**	**7543**	**6527**

**Total (exc. MARS)**	**16908**	**15433**	**7543**	**6527**

**Total/d (exc. MARS)**	**636**	**712**	**428**	**498**

### Direct medical costs during follow-up

Average direct medical costs per patient of the MARS group were 8493 EUR over a follow-up period of 3 years. Most important cost components were inpatient acute care, rehabilitation and drug use. After adjustment of the costs for the mortality differences by excluding the non-survivors mean treatment costs per year per (surviving) patient of 5827 EUR in the MARS group and 12092 EUR in the control group were caused. Two trends could be observed in this subgroup: (1) Over the period of three years as well as in every single year the treatment of patients after MARS caused lower costs compared to the controls. (2) The longer the duration of the follow-up the more the treatment costs decreased.

Table 4 shows the health care expenses of the patients during the follow-up in detail.

**Table 4:** Direct medical cost during 3 years follow-up in EUR 2002

	**MARS**		**Controls**	
	Mean	SD	Mean	SD
Inpatient acute care	3911	5583	3137	5418
Inpatient rehab	2010	5516	766	3183
GP	310	452	192	366
Specialist	510	1030	158	482
Other ambul. Care	238	31	248	414
Nursing	0	-	0	-
Drugs (ambul.)	1360	1745	629	1012
Other	130	194	63	144

**Total (3a)**	**8493**	**10268**	**5194**	**7176**

### Influence of relevant parameter on direct medical costs

The influence of relevant socio-demographic and clinical parameter on the total direct medical costs was tested: Besides MARS a significant influence was seen only for creatinine indicating that an additional renal failure respectively multiorgan failure was responsible for higher costs. Other parameters expressing the severity of ACLF like CHILD-Score or the grade of hepatic encephalopathy did not significantly change the resource use. The results are shown in detail in table 5

**Table 5:** Influence of relevant parameter on direct medical costs

***Parameter***	***p-value***	***Parameter***	***p-value***	***Parameter***	***p-value***
MARS	0.000*	HE-Grade	0.400	Quick	0.164
Sex	0.604	Child-Score	0.886	Ren. Dialysis	0.072
Age	0.938	Platelets.	0.574	CRP	0.168
Bilirubin	0.260	Creatinine	0.004*	Fibrinogen	0.361

* significant difference (p < 0.05) tested with Mann-Whithney-U Test or OLS-Regression

### Incremental cost-effectiveness ratios

Treatment with MARS led to an incremental gain of survival of 285d per patient over the period of 3 years. In relation to the additional costs of 27243EUR per patient over the same time period an incremental cost-effectiveness ratio of 31448 EUR per life-year gained could be determined. Weighing the survival time using the estimated group-specific health-related quality of life of the patients the incremental costs per QALY gained were 47171 EUR.

## Discussion

The artificial liver support system MARS is a new emerging innovative medical technology used for the treatment of patients with liver failure. Although in first randomized clinical trials the short-time clinical efficacy of the technology in patients with ACLF has been demonstrated[11,12,21], the technology is not yet included in routine care and is just about being clinically evaluated for a broader use.

For the first time prospectively collected controlled-trial data on survival, costs and cost-effectiveness over a time horizon of three years are presented.

The cost-effectiveness of MARS expressed in costs per life-year gained is in a reasonable range compared to other reimbursed medical technologies and the implicit cutoffs discussed e.g. in UK or USA [22,23]. This interpretation is seen as conservative, because a prolongation of the time horizon from 3 years to the whole lifetime of the patients might well improve the cost-effectiveness of MARS by decreasing the costs per life year gained. Furthermore a more frequent use of the technology in a routine care setting would probably lead to better clinical outcomes as physicians gain more experience and by scale effect also lower retail prices.

In clinical trials the short-time mortality, e.g. expressed as survival-rate after 30 days, was chosen as a relevant outcome parameter[4,11,12]. A period of one month seems to be much too short to calculate costs respectively the cost-effectiveness of a medical technology, which possibly affects the rest of the life of the patients. For the first time the direct medical costs for the treatment of patients with ACLF over a period of three year are described. Also over this longer period of time the initial hospital stay is crucial for the costs of the whole treatment. The costs of the initial hospital stay itself are mainly influenced by the use of MARS. The pure intervention costs of MARS are certainly high (nearly 15000 EUR per patient) and including all hospital resources used during the acute inpatient treatment of ACLF the costs add up to further 17000 EUR per patient treated with MARS.

The chances for the hospital to completely compensate the costs of MARS by reducing the number of inpatient days are low. Therefore a strategy additionally using MARS can hardly dominate standard care in terms of cost-effectiveness from a hospital's point of view. On the longer run the direct medical costs are still significantly higher compared to patients with ACLF not treated with MARS, whether tested with parametetric or simple non-parametric tests. As there were no significant differences in baseline characteristics no adjustments for these variables were performed and the costs were directly included into cost-effectiveness calculations as mean values.

The results regarding survival are highly significant, but they should not be over estimated. For the first time in a controlled study design the three-year survival after MARS is described. Previous randomized studies with much smaller sample sizes showed significant differences in survival probabilities after 30d [4,11,12], but could not reproduce the results after one year (possibly because the studies have not been powered for). A randomized design investigating the cost-effectiveness of MARS could not be realized due to ethical and practical reasons like limited ressources. Larger randomized controlled studies with hard clinical endpoints are missing so far and the results of the present cohort study have to be confirmed by future trials[21,24]. The danger of a selection bias is definitely present in every non-randomized clinical cohort study. There were no relevant differences between controls and patients of the intervention group at baseline concerning classical confounder like age, sex, and severity of the disease (see also table 1), but other possible confounders could be controlled only by randomization. All patients over a defined period of time were included in the study consecutively and therefore any selection by purpose from the investigators seems to be impossible. The Rostock University Hospital is a centre of excellence for the treatment of patients with MARS and one of the few hospitals worldwide where a larger number of patients was treated with this technology.

The inclusion and exclusion criteria for this study were chosen according to the indication criteria for MARS. They are identical with those of the previous clinical trials. According to the treating physicians the decision for MARS was not made because of the subjective individual prognosis of the patient, but according to the availability of MARS treatment and expert personal. Nevertheless the disadvantages of a non-randomized design are present and it can not be denied that the survival results, which are quite favorable for MARS, could overestimate the outcome gained due to a selection bias or confounding.

From a health economic point of view there are also some limitations. At present only by model calculations like medical decision analysis techniques, e.g. Markov modelling, the time horizon could be extended, but as long as long-time survival and outcome data are missing model calculations will bare a lot of uncertainty. A time horizon of 3 years and a sample size like it could be realized in this study is the maximum to achieve in an empirical analysis of a young emerging technology like MARS.

We did not include direct non-medical costs like travel expenses, costs for child care or other out-of-pocket costs due to a lack of valid data. We also did not include indirect costs of productivity loss. But the indirect costs were considered as neglectable, because the vast majority of the patients already received an invalidity pension and only two patients of the MARS group were able to return to a regular part-time work during the follow-up period.

The preliminary results on health related quality of life using EQ-5D and SF-12D have been published previously[13]. We continued the measurements asking all surviving patients in standardized telephone interviews. The results after two and three years exactly reproduced the previous results after 6 months and one year confirming that there is no difference in the health related quality of life between patients after ACLF treated with or without using MARS. These measurements can not be interpreted as a separate quality of life study e.g. because of the missing of baseline data. But they are seen as a good approximation for the purpose of weighting the life-years gained to calculate QALYs. For valid results on health related quality of life further studies have to be awaited.

We excluded the few patients considered for liver transplantation – alcohol abuse is a contra-indication for organ transplantation – because the high treatment costs of these few patients could extremely influence the costs and cost-effectiveness of the treatment alternatives. Therefore the effect of the use of MARS for bridging to transplantation could not be included in this study.

## Conclusion

The institutional frame of the German health care system makes it even more difficult to calculate the cost effectiveness of innovative, high-priced medical devices with a sufficient sample size in a real-life setting than it is compared to pharmaceutical innovations. In conclusion the results of the first economic evaluation of MARS imply that at least for the group of patients with ACLF and a documented alcohol abuse a treatment with the artificial liver support system MARS results in a higher survival probability accompanied by reasonable costs per life-year gained and costs per QALY. The results of further sufficiently powered studies have to be waited for and the need for a randomized control study including economic aspects can only be underlined again.

## Competing interests

The study was partly supported by an unrestricted grant from Teraklin, Rostock, Germany.

## Authors' contributions

The author has made substantial contributions to conception and design, and acquisition of data, and analysis and interpretation of data; 2) has been involved in drafting the manuscript or revising it critically for important intellectual content; and 3) has given final approval of the version to be published.
